# Bioelectrical Regulation of Vascular Endothelial Function in Atherosclerosis

**DOI:** 10.3390/biom16071000

**Published:** 2026-07-09

**Authors:** Julienne Marie Custodio, Jianhua J. Liu, Lu Zhang, Liang Hong

**Affiliations:** 1 Division of Cardiology, Department of Medicine, University of Illinois Chicago, Chicago, IL 60612, USA; jcust2@uic.edu; 2 Department of Pathology, University of Illinois Chicago, Chicago, IL 60612, USA; jliu261@uic.edu; 3 Center for Cardiovascular Research, University of Illinois Chicago, Chicago, IL 60612, USA; 4 Department of Physiology and Biophysics, University of Illinois Chicago, Chicago, IL 60612, USA; 5 Department of Biomedical Engineering, University of Illinois Chicago, Chicago, IL 60612, USA

**Keywords:** endothelium, endothelial cells, atherosclerosis, bioelectrical signal, ion channel

## Abstract

Atherosclerosis is a chronic inflammatory disease characterized by progressive vascular dysfunction and remains a leading cause of cardiovascular morbidity and mortality worldwide. Endothelial dysfunction is an early and critical event in atherogenesis, contributing to inflammation, leukocyte recruitment, plaque progression, and thrombotic complications. Increasing evidence indicates that vascular endothelial cells use bioelectrical signaling mechanisms to integrate mechanical, metabolic, and inflammatory cues and maintain vascular homeostasis. At the core of these regulatory networks are ion channels and transporters, which coordinate membrane potential, ionic fluxes, calcium homeostasis, redox balance, and downstream biochemical signaling to regulate endothelial function in vascular health and disease. Dysregulation of these pathways may promote oxidative stress, inflammation, endothelial senescence, apoptosis, endothelial-to-mesenchymal transition, and impaired vasodilatory function, thereby contributing to atherosclerotic progression. Understanding how ion channels regulate endothelial function may provide important insights into atherosclerosis and facilitate the development of novel therapeutic strategies aimed at restoring endothelial homeostasis and reducing cardiovascular risk.

## 1. Introduction

Atherosclerosis is a chronic inflammatory disease of the arterial wall and the underlying cause of most cardiovascular diseases, including myocardial infarction, stroke, and peripheral arterial disease [1,2,3]. The vascular endothelium, a monolayer of cells lining the inner surface of blood vessels, plays a pivotal role in maintaining vascular homeostasis by regulating vascular tone, barrier integrity, thrombosis, inflammation, and leukocyte trafficking [4]. These endothelial functions are regulated not only by classical biochemical signaling pathways but also by bioelectrical mechanisms involving membrane potential, ionic fluxes, intracellular calcium dynamics, and ion transporter activity. Endothelial dysfunction is widely recognized as one of the earliest events in atherogenesis and precedes the development of overt vascular lesions [5]. In the presence of cardiovascular risk factors such as hyperlipidemia, hypertension, diabetes, smoking, and disturbed shear stress, endothelial cells undergo pathological activation characterized by impaired nitric oxide signaling, excessive oxidative stress, increased vascular permeability, and elevated expression of adhesion molecules [6,7,8]. These alterations disrupt vascular homeostasis and compromise the barrier and anti-inflammatory functions of the endothelium, creating a pro-atherogenic microenvironment. Consequently, the deposition and accumulation of atherogenic lipoproteins within the vessel wall are enhanced, while increased expression of adhesion molecules facilitates the recruitment, adhesion, and transmigration of circulating inflammatory cells into the intima, thereby initiating atherosclerotic lesion formation.

As atherosclerosis progresses, endothelial cells continue to play active roles in vascular inflammation, leukocyte trafficking, plaque remodeling, and neovascularization. Persistent endothelial dysfunction further contributes to plaque growth and instability by promoting oxidative stress, inflammatory signaling, and thrombotic responses, ultimately increasing the risk of plaque rupture and adverse cardiovascular events [5]. Therefore, elucidating the molecular mechanisms that regulate endothelial function is critical for understanding the pathogenesis of atherosclerosis and for informing the development of potential therapeutic strategies. Beyond classical biochemical pathways, increasing attention has been directed toward bioelectrical mechanisms that shape endothelial responses to mechanical, metabolic, and inflammatory stimuli, with ion channels as key regulatory components.

## 2. Endothelial Ion Channels

Ion channels are integral membrane proteins that mediate the selective transport of ions across cellular membranes and constitute fundamental components of cellular bioelectrical signaling. Dysregulation of ion channel function has been linked to numerous human diseases [9,10,11,12,13,14,15], highlighting their critical roles in physiology and pathophysiology. Although endothelial cells do not generate classical action potentials, they possess a diverse array of ion channels that integrate mechanical, metabolic, and inflammatory stimuli to regulate intracellular calcium dynamics, membrane potential, mechanotransduction, and intercellular communication [16]. By coordinating these bioelectrical and biochemical signaling pathways, endothelial ion channels modify nitric oxide production, redox homeostasis, vascular permeability, inflammatory responses, and cell survival, thereby serving as key determinants of vascular homeostasis and endothelial function [16,17,18].

Endothelial cells express a diverse repertoire of ion channels, channel-like proteins, and transport-related regulators [16], including transient receptor potential (TRP) channels, potassium channels, Piezo mechanosensitive channels, aquaporins (AQPs), epithelial sodium channels (ENaC), chloride channels, pannexins, and intracellular calcium-release channels. These channels respond to a broad spectrum of chemical, mechanical, metabolic, and inflammatory stimuli, allowing endothelial cells to continuously adapt to changes in the vascular microenvironment. Importantly, accumulating evidence indicates that dysregulation of endothelial ion channels contributes to oxidative stress, inflammation, endothelial senescence, apoptosis, ferroptosis, endothelial-to-mesenchymal transition (EndMT), and impaired vasodilatory function during atherosclerosis.

Recent advances have revealed that endothelial ion channels function not as isolated signaling molecules but as components of integrated bioelectrical signaling networks that coordinate vascular responses to hemodynamic and metabolic stress [19]. Therefore, characterization of endothelial ion channel pathways will provide novel insights into the pathogenesis of atherosclerosis and identify new therapeutic targets for the treatment of endothelial dysfunction and atherosclerosis.

## 3. Endothelial TRP Channels and Atherosclerosis

Transient receptor potential (TRP) channels comprise a large superfamily of nonselective cation channels that function as important regulators of endothelial calcium signaling, mechano-transduction, oxidative stress responses, inflammation, and cell survival [20]. Accumulating evidence indicates that dysregulation of endothelial TRP channels contributes to multiple stages of atherosclerosis, including endothelial activation, leukocyte recruitment, oxidative injury, apoptosis, senescence, and vascular remodeling. Although individual TRP subfamilies exert distinct effects, they collectively influence endothelial homeostasis and plaque progression through interconnected signaling pathways [21]. The following sections summarize current knowledge regarding the roles of endothelial TRPC, TRPV, TRPM, and TRPML channels in atherosclerosis.

### 3.1. TRPC

Among the TRP canonical (TRPC) family members, TRPC3 and TRPC6 have been most extensively implicated in the regulation of endothelial dysfunction and atherosclerosis progression. Endothelial TRPC channels influence multiple pro-atherogenic pathways, including leukocyte recruitment, oxidative stress, endothelial barrier dysfunction, and plaque neovascularization, positioning them as potential therapeutic targets in vascular disease [22].

TRPC3 plays a key role in regulating endothelial inflammatory activation [23,24,25]. Studies in human coronary artery endothelial cells demonstrated that TRPC3-dependent Ca^2+^ influx is required for NF-κB activation, vascular cell adhesion molecule-1 (VCAM-1) expression, and monocyte adhesion, which represent critical early events in atherogenesis. Endothelial-specific overexpression of TRPC3 in *Apoe^−/−^* mice accelerated lesion progression and was associated with increased endothelial VCAM-1 expression, enhanced NF-κB signaling, and greater macrophage accumulation within plaques, providing direct in vivo evidence for a pro-atherogenic role of endothelial TRPC3 [23]. In addition, increased TRPC3 activity was shown to promote endoplasmic reticulum stress, endothelial apoptosis, and vascular dysfunction under hyperlipidemic conditions [24].

Similar to TRPC3, TRPC6 contributes to endothelial dysfunction during atherosclerosis [26,27]. In *Apoe^−/−^* mice and ox-LDL-treated endothelial cells, TRPC6 expression is upregulated, whereas expression of the protective microRNA miR-26a is reduced. Restoration of miR-26a suppresses TRPC6 expression, inhibits mitochondrial apoptotic signaling, and protects endothelial cells from ox-LDL-induced injury. Conversely, TRPC6 overexpression abolishes the protective effects of miR-26a, supporting a pathogenic role for TRPC6 in endothelial apoptosis and atherosclerosis progression [26].

### 3.2. TRPV

Members of the TRP vanilloid (TRPV) channels exert diverse effects on endothelial function during atherosclerosis. Current evidence suggests that TRPV1 and TRPV6 predominantly confer endothelial protection through modulation of inflammation, oxidative stress, mechanotransduction, and cell survival pathways.

Among these channels, TRPV1 is the most extensively studied and generally exhibits anti-atherogenic properties. In the cardiovascular system, TRPV1 activation has been shown to protect endothelial cells, inhibit vascular inflammation, and attenuate the progression of atherosclerosis [28]. Endothelial dysfunction is a critical initiating event in atherogenesis. Activation of TRPV1 by agonists such as capsaicin and evodiamine alleviates endothelial injury and suppresses vascular inflammation. Wang et al. demonstrated that TRPV1 activation by evodiamine significantly reduced atherosclerotic lesion size in *Apoe^−/−^* mice [29]. TRPV1 activation inhibited Rho-associated coiled-coil containing protein kinase (ROCK) signaling and reduced endothelial inflammation. Pharmacological blockade or silencing of TRPV1 abolished these protective effects, highlighting the role of endothelial TRPV1 signaling in maintaining vascular homeostasis [29]. Recent studies found that TRPV1 deficiency increased expression of interferon-stimulated gene 15 (ISG15), which activated the p53/p21 signaling pathway, promoting endothelial cell senescence underlying atherogenesis, suggesting that TRPV1 protects against atherosclerosis by suppressing endothelial aging through the ISG15-p53 axis [30].

In contrast to TRPV1, the role of TRPV4 appears more complex and context-dependent [31,32,33,34,35]. Early studies showed that pharmacological activation of TRPV4 mimics atheroprotective laminar shear stress signaling. TRPV4 activation stimulates Ca^2+^ influx, leading to activation of endothelial nitric oxide synthase (eNOS), enhanced NO production, and attenuation of atherosclerotic plaque formation in *Apoe^−/−^* mice [32]. These findings established TRPV4 as a regulator of endothelial homeostasis and vascular protection. More recent work has highlighted the role of TRPV4 as a vascular mechanosensor during disease progression. TRPV4-mediated mechanotransduction regulates matrix stiffness-induced EndMT, a process increasingly implicated in plaque formation and vascular remodeling. Genetic deletion or pharmacological inhibition of TRPV4 suppresses EndMT and prevents acquisition of mesenchymal phenotypes in aortic endothelial cells [33]. In addition, TRPV4 participates in membrane tension sensing and inflammatory signaling. In endothelial cells exposed to oxidized low-density lipoprotein (ox-LDL), TRPV4-dependent mechanosignaling contributes to mitochondrial dysfunction, NLRP3 inflammasome activation, and pyroptosis, whereas modulation of TRPV4 activity attenuates endothelial inflammation and slows atherosclerotic progression [34].

In addition to TRPV1 and TRPV4, emerging evidence suggests that TRPV6 may also contribute to endothelial protection. Zheng et al. reported that TRPV6 expression is significantly reduced in *Apoe^−/−^* atherosclerotic mice and in ox-LDL-treated human umbilical vein endothelial cells (HUVECs), suggesting that loss of TRPV6 may contribute to endothelial dysfunction during atherogenesis. Functional studies demonstrated that TRPV6 overexpression increased endothelial cell viability while suppressing apoptosis and inflammatory cytokine production, including TNF-α, IL-6, and IL-1β. The findings suggest that endothelial TRPV6 may exert an atheroprotective role by limiting inflammation and apoptosis. However, evidence remains limited to cellular and experimental models, and further studies are required to determine whether targeting TRPV6 can effectively attenuate atherosclerosis in vivo [36].

### 3.3. TRPM

The transient receptor potential melastatin (TRPM) family has emerged as an important regulator of endothelial responses to oxidative and metabolic stress during atherosclerosis. Among them, TRPM2 and TRPM4 have been implicated in endothelial inflammation, oxidative stress signaling, and vascular injury [37,38].

TRPM2 functions as a redox sensor linking oxidative stress to inflammatory signaling. TRPM2 is expressed in endothelial cells and is activated by reactive oxygen species (ROS), leading to intracellular Ca^2+^ influx and amplification of inflammatory signaling. Using hypercholesterolemic mouse models, Zhang et al. demonstrated that genetic deletion of TRPM2 markedly reduced atherosclerotic plaque formation, vascular ROS accumulation, and expression of inflammatory factors [37]. Furthermore, TRPM2 deficiency attenuated H_2_O_2_-induced Ca^2+^ entry and reduced inflammatory responses in primary arterial endothelial cells, suggesting that TRPM2 provides a mechanistic link between oxidative stress, calcium signaling, and endothelial activation during atherogenesis. These findings support a pro-atherogenic role of endothelial TRPM2 signaling.

More recently, TRPM4 has been identified as another contributor to endothelial dysfunction and plaque instability [38]. TRPM4 expression is significantly increased in the endothelium of atherosclerotic *Apoe^−/−^* and *Ldlr^−/−^* mice and is upregulated in endothelial cells exposed to ox-LDL. Pharmacological inhibition of TRPM4 with 9-phenanthrol or TRPM4 knockdown improves endothelial viability and suppresses excessive autophagy and apoptosis. Importantly, TRPM4 inhibition also enhances plaque stability in vivo by reducing necrotic core formation and increasing smooth muscle cell content within plaques.

Collectively, endothelial TRP channels represent a diverse but functionally interconnected group of calcium-permeable and cation-conducting channels that regulate multiple stages of atherogenesis. TRPC3 and TRPC6 are generally associated with pro-atherogenic endothelial activation, leukocyte adhesion, ER stress, apoptosis, and lipid-induced injury, whereas TRPV1 and TRPV6 appear to exert predominantly protective effects by limiting inflammation, oxidative stress, and apoptosis. TRPV4 and TRPM channels illustrate the context-dependent nature of TRP signaling. TRPV4 may support endothelial homeostasis through shear stress-dependent Ca^2+^ influx, eNOS activation, and NO production, but under pathological mechanical or lipid stress it can also contribute to EndMT, inflammasome activation, pyroptosis, and vascular remodeling. Similarly, current evidence supports TRPM2 and TRPM4 as important regulators of oxidative stress- and lipid-induced endothelial injury. These findings suggest that TRP channels do not act as isolated regulators but participate in broader endothelial signaling networks that integrate mechanical forces, lipid stress, ROS generation, calcium signaling, inflammatory transcriptional programs, and cell survival pathways. However, the strength of evidence varies across TRP subfamilies. While TRPC3, TRPC6, TRPV1, TRPV4, TRPM2, and TRPM4 are supported by in vitro and preclinical animal studies, human endothelial-specific evidence and clinical validation remain limited. Future studies using human vascular tissues, single-cell and spatial transcriptomic approaches, endothelial-specific genetic models, and selective pharmacological modulators will be needed to clarify the translational relevance of targeting TRP channels in atherosclerosis.

## 4. Endothelial Potassium Channels in Atherosclerosis

Potassium (K^+^) channels are essential determinants of endothelial membrane potential and play fundamental roles in regulating calcium signaling, nitric oxide production, and vascular homeostasis. Multiple K^+^ channel families are expressed in endothelial cells, including calcium-activated potassium (K_Ca_) channels, ATP-sensitive potassium (K_ATP_) channels, and voltage-gated potassium (K_v_) channels. Although these channels are activated by distinct stimuli, they converge functionally to promote endothelial hyperpolarization and vasodilatory signaling. Increasing evidence indicates that dysregulation of endothelial K^+^ channels contributes to impaired vascular relaxation, oxidative stress, inflammation, and endothelial dysfunction during atherosclerosis [39].

### 4.1. K_Ca_

Calcium-activated potassium (K_Ca_) channels are important regulators of endothelial membrane potential, intracellular calcium signaling, and vascular tone. Among the K_Ca_ family, the small-conductance K_Ca_2.3 and intermediate-conductance K_Ca_3.1 channels are highly expressed in endothelial cells. Increasing evidence suggests that dysfunction of endothelial K_Ca_ channels contributes to impaired vascular relaxation and endothelial dysfunction during atherosclerosis [40,41,42,43]. Early studies in atherosclerotic mouse models found that endothelial-dependent vasodilation is impaired in diseased vessels and is accompanied by altered potassium channel activity. In *Apoe^−/−^Ldlr^−/−^* double-knockout mice, pharmacological blockade of K_Ca_ channels with tetraethylammonium or charybdotoxin abolished nitric oxide (NO)-dependent vasomotor activity, suggesting that enhanced K_Ca_ channel opening acts as a compensatory mechanism to preserve vascular function under atherosclerotic conditions [40]. The findings showed a functional interaction between endothelial NO signaling and K_Ca_ channel activity in diseased arteries. Consistent with these discoveries, studies in atherogenic arteries found that K_Ca_ channels participate in endothelial-dependent vasorelaxation pathways and contribute to the maintenance of vascular homeostasis under pathological conditions [41]. More recently, endothelial K_Ca_2.3 and K_Ca_3.1 channels have emerged as attractive therapeutic targets. In an *Apoe^−/−^* mouse model of atherosclerosis, chronic administration of the K_Ca_2.3/K_Ca_3.1 activator SKA-31 restored endothelium-dependent relaxation to levels observed in healthy control mice. Notably, SKA-31 improved endothelial function despite having no significant effect on atherosclerotic plaque burden, suggesting that enhancement of endothelial K_Ca_ channel activity primarily targets vascular dysfunction [42].

### 4.2. K_ATP_

In contrast to K_Ca_ channels, ATP-sensitive potassium (K_ATP_) channels function primarily as metabolic sensors that couple cellular energy status to endothelial electrical activity and vascular function. In endothelial cells, K_ATP_ channels are primarily composed of K_ir_6.1 pore-forming subunits and sulfonylurea receptor (SUR) regulatory subunits and contribute to endothelial hyperpolarization, calcium signaling, NO production, and vascular homeostasis [44]. Emerging evidence suggests that endothelial K_ATP_ channels show a protective role against atherosclerosis. Endothelial-specific deletion of Kir6.1 abolished endothelial K_ATP_ currents and impaired calcium influx in response to channel activation, indicating a role of the channel in endothelial signaling and mediator release. Functional studies demonstrated that activation of endothelial K_ATP_ channels promotes endothelium-dependent vasorelaxation [44]. More importantly, endothelial K_ATP_ channel deficiency accelerated atherosclerotic lesion formation. In *Apoe^−/−^* mice, endothelial-specific deletion of K_ir_6.1 significantly increased aortic plaque burden, particularly within the aortic arch, and impaired vascular reactivity in resistance arteries following high-fat diet exposure [44]. These findings indicate that endothelial K_ATP_ channels preserve endothelial integrity and protect against diet-induced vascular injury and atherogenesis. In addition, other studies highlighted the broader cardioprotective actions of mitochondrial K_ATP_ channels, which reduce oxidative stress and limit endothelial apoptosis linked to atherosclerotic progression [45]. Together, these studies support a vascular protective role for endothelial K_ATP_ channels in maintaining endothelial integrity and limiting atherosclerotic lesion development.

### 4.3. K_v_

Voltage-gated potassium (K_v_) channels represent another important class of endothelial potassium channels that regulate membrane excitability and vasomotor responses. Activation of endothelial K_v_ channels promotes membrane hyperpolarization, facilitates calcium influx, and supports the production of vasodilatory mediators such as NO. Altered K_v_ channel activity has been shown to contribute to endothelial dysfunction and abnormal vascular reactivity during disease progression [40,46]. In *Apoe^−/−^Ldlr^−/−^* double-knockout mice, endothelium-dependent relaxation was significantly impaired, accompanied by abnormalities in vascular rhythmic activity and potassium channel regulation. Pharmacological inhibition of K_v_ channels with 4-aminopyridine (4-AP) increased basal vascular tone in atherosclerotic vessels, indicating reduced basal K_v_ channel activity and increased susceptibility to membrane depolarization. These findings suggest that diminished K_v_ channel function contributes to altered calcium handling and impaired endothelial NO signaling during atherosclerosis [40]. Additional evidence supports a compensatory role for K_v_ channels in preserving endothelial function under pro-atherogenic conditions. In *Apoe^−/−^* mice with endothelial-specific endothelin-1 overexpression, endothelium-dependent relaxation was maintained despite accelerated atherosclerosis. Mechanistic studies showed enhanced activation of 4-AP-sensitive K_v_ channels during acetylcholine-induced relaxation, indicating that increased K_v_-dependent hyperpolarization may compensate for impaired NO signaling and help preserve vasodilatory responses [46].

Collectively, endothelial K^+^ channels function as central regulators of membrane potential and vasodilatory signaling during vascular homeostasis and atherosclerosis. Although K_Ca_, K_ATP_, and K_v_ channels are activated by different stimuli, they converge on a common functional outcome: endothelial hyperpolarization, which supports calcium entry, eNOS activation, NO production, and endothelium-dependent relaxation. Current evidence suggests that K_Ca_ channels may act as compensatory regulators that help preserve vasomotor function in atherosclerotic vessels, while pharmacological activation of K_Ca_2.3/K_Ca_3.1 improves endothelial function and vasodilation without necessarily reducing plaque burden. Endothelial K_ATP_ channels appear to exert broader protective effects by coupling metabolic status to endothelial electrical activity, vascular reactivity, oxidative stress control, and resistance to diet-induced atherogenesis. K_v_ channels may similarly contribute to basal membrane potential regulation and, in some disease settings, compensate for impaired NO signaling to maintain vasodilatory responses. These findings support the concept that endothelial K^+^ channels are not merely passive determinants of membrane potential but are functionally integrated with calcium signaling, NO bioavailability, redox regulation, and vascular inflammatory responses. However, most available evidence remains derived from genetic or pharmacological studies in atherosclerotic mouse models and isolated vessels, and human endothelial-specific validation is still limited. Further studies are needed to determine whether selective modulation of endothelial K^+^ channel subtypes can improve vascular function and reduce cardiovascular risk in clinically relevant settings.

## 5. Other Channels in Endothelial Cells in Atherosclerosis

### 5.1. Piezo1

Piezo1 is a mechanically activated, nonselective cation channel that functions as a key endothelial mechanosensor, converting hemodynamic forces into intracellular biochemical signals. Because atherosclerotic lesions preferentially develop at arterial branches and curvatures exposed to disturbed flow and oscillatory shear stress, increasing attention has focused on the role of endothelial Piezo1 in atherogenesis [35,47,48,49,50,51]. Recent studies have demonstrated that Piezo1 expression is significantly elevated in atherosclerotic plaques, disturbed-flow regions, and endothelial cells exposed to oscillatory shear stress or ox-LDL, indicating its involvement in endothelial dysfunction and vascular inflammation [52,53,54,55].

Mechanistically, Piezo1 activation promotes Ca^2+^ influx, which triggers multiple downstream pro-atherogenic signaling pathways. Several studies identified YAP/TAZ as a critical downstream effector of Piezo1. Activation of Piezo1 induces YAP/TAZ nuclear translocation and transcriptional activity, resulting in increased expression of inflammatory mediators including NF-κB, TNF-α, VCAM-1, and ICAM-1, thereby promoting endothelial inflammation and leukocyte recruitment [53,54,55]. In addition, disturbed flow activates the Piezo1-Ca^2+/^CaM/CaMKII-FAK/Src-YAP signaling cascade, amplifying endothelial inflammatory responses and accelerating plaque development [55].

Beyond inflammation, Piezo1 influences epigenetic and vascular remodeling pathways. Piezo1-mediated mechanotransduction promotes expression of the histone demethylase KDM5B under disturbed flow conditions, thereby facilitating endothelial inflammatory gene expression and atherosclerotic plaque formation [52]. Furthermore, Piezo1 activation enhances Talin1-YAP signaling and contributes to endothelial inflammation under oscillatory shear stress [54]. Recent studies also suggest that excessive Piezo1 activity impairs intraplaque neovessel maturation through YAP- and angiopoietin-1-dependent mechanisms, thereby increasing plaque vulnerability [56].

Importantly, pharmacological inhibition or genetic deletion of Piezo1 consistently attenuates endothelial inflammation, reduces atherosclerotic plaque burden, and improves vascular function in experimental models [53,55,57,58]. Collectively, these findings establish endothelial Piezo1 as a central mechanotransducer linking disturbed hemodynamic forces to inflammation, endothelial dysfunction, and plaque progression, highlighting Piezo1 as a promising therapeutic target for atherosclerotic cardiovascular disease.

### 5.2. AQP

Aquaporins (AQPs) are membrane channel proteins that facilitate the transcellular transport of water and small solutes regulating cellular volume, permeability, metabolism, and redox homeostasis. Among the AQP family, AQP1 is the major endothelial water channel and an important regulator of vascular homeostasis and cardiovascular health [59,60]. Recent studies suggest that endothelial AQPs contribute to several processes involved in atherosclerosis, including endothelial dysfunction, inflammation, cell death, and plaque remodeling.

AQP1 is abundantly expressed in vascular endothelial cells and mediates transcellular water transport across the endothelium. In cultured human endothelial cells, a pro-atherogenic hypomethylating environment reduced AQP1 expression and impaired endothelial water permeability, whereas inflammatory stimulation with TNF-α similarly suppressed AQP1 expression [59]. These findings suggest that endothelial dysfunction and inflammatory activation are associated with AQP1 downregulation and altered endothelial barrier function. Moreover, theoretical modeling studies have proposed that endothelial AQP1-mediated water transport influences transmural fluid flow and subendothelial LDL handling, potentially affecting the earliest stages of atherogenesis [6]. Evidence from animal and human studies further supports a protective role for AQP1 in atherosclerosis. AQP1 is highly expressed in the microvasculature of human atherosclerotic plaques, as well as in murine atherosclerotic lesions. Notably, AQP1-deficient *Apoe^−/−^* mice exhibited enhanced angiotensin II-induced atherosclerotic lesion development, indicating that normal AQP1 function affords cardiovascular protection [60]. More recently, transcriptomic, spatial transcriptomic, and functional studies demonstrated that AQP1 is highly enriched in endothelial cells within atherosclerotic lesions. Overexpression of AQP1 suppressed endothelial necroptosis, and promoted endothelial cell survival, suggesting that AQP1 may stabilize plaques by limiting necroptotic cell death [61].

In addition to AQP1, other AQP family members may also influence atherosclerosis. AQP3, AQP5, AQP8, and AQP9 facilitate hydrogen peroxide transport and are increasingly recognized as regulators of cellular oxidative stress. In *Apoe^−/−^* mice fed an atherogenic diet, AQP5 expression was significantly increased and associated with enhanced systemic inflammation and atherosclerotic burden, suggesting a potential contribution of AQPs to cardiometabolic dysfunction and vascular disease [62].

### 5.3. ENaC

The epithelial sodium channel (ENaC) is expressed in vascular endothelial cells, where it regulates endothelial stiffness and vascular function. Emerging evidence indicates that endothelial ENaC contributes to atherogenesis by promoting endothelial dysfunction and vascular inflammation. In *Ldlr^−/−^* mice, high-fat diet feeding increased endothelial ENaC activity, impaired endothelium-dependent relaxation, and accelerated atherosclerotic lesion formation, whereas treatment with the ENaC blocker benzamil markedly reduced plaque burden, improved endothelial function, and suppressed vascular inflammation [63]. Mechanistically, ox-LDL activated endothelial ENaC, leading to increased expression of pro-inflammatory cytokines and adhesion molecules, promoting leukocyte recruitment and atherosclerosis progression [63]. In addition, studies found that ENaC activities blocked with amiloride partially attenuated atherosclerosis and endothelial VCAM-1 expression, supporting a role for ENaC-mediated endothelial inflammation in plaque development [7]. These findings suggest endothelial ENaC as a pro-atherogenic ion channel that links metabolic and inflammatory stimuli to endothelial dysfunction and vascular inflammation.

### 5.4. HCN

Hyperpolarization-activated cyclic nucleotide-gated (HCN) channels, which generate the pacemaker current (I_f_), have emerged as potential modulators of vascular pathology through their effects on heart rate and endothelial function. In *Apoe^−/−^* mice, pharmacological inhibition of I_f_ channels with ivabradine restored endothelial-dependent vasorelaxation, enhanced eNOS activity, and reduced systemic inflammatory cytokine levels. These changes were accompanied by attenuation of atherosclerosis and improved collateral vessel growth [64]. Although direct effects of ivabradine on cultured endothelial cells were not observed, these findings suggest that HCN channel modulation exerts anti-atherosclerotic actions by improving endothelial function, reducing vascular inflammation, and restoring NO-dependent vascular homeostasis in atherosclerotic vessels.

### 5.5. IP3R1

Inositol 1,4,5-trisphosphate receptor 1 (IP3R1) is a major intracellular Ca^2+^-release channel localized primarily on the endoplasmic reticulum membrane of vascular endothelial cells [65]. By mediating IP3-dependent Ca^2+^ release from the endoplasmic reticulum, IP3R1 regulates intracellular Ca^2+^ dynamics and contributes to endothelial Ca^2+^ homeostasis. Recent studies showed that oxidized LDL and cholesterol crystals promote endothelial dysfunction by inducing epsin-mediated ubiquitination and proteasomal degradation of IP3R1, resulting in impaired Ca^2+^ signaling and enhanced endothelial inflammation [66]. Endothelial-specific deletion of IP3R1 accelerated atherosclerotic lesion formation, whereas stabilization of IP3R1 preserved Ca^2+^ homeostasis, reduced expression of adhesion molecules such as ICAM-1 and VCAM-1, and attenuated leukocyte recruitment [66]. IP3R1 expression was reduced in atherosclerotic lesions, supporting an atheroprotective role for endothelial IP3R1-mediated Ca^2+^ signaling. The findings identify endothelial IP3R1 as an important regulator of vascular homeostasis and a potential therapeutic target in atherosclerosis.

### 5.6. Pannexin 1

Pannexin 1 (Panx1) is an ATP-permeable membrane channel expressed in vascular endothelial cells and lymphatic endothelial cells, where it regulates purinergic signaling, inflammation, and vascular homeostasis. Studies showed that endothelial Panx1 plays a protective role in atherosclerosis. In *Apoe^−/−^* mice, endothelial-specific deletion of Panx1 increased atherosclerotic lesion formation without altering serum cholesterol levels, indicating that Panx1-mediated signaling limits plaque development through local vascular mechanisms rather than systemic lipid regulation [67]. Panx1 is highly expressed in endothelial cells and upregulated in macrophage foam cells within atherosclerotic lesions, supporting its involvement in vascular inflammatory responses [67].

Recent studies extended the findings to the lymphatic vasculature. Lymphatic endothelial cell-specific deletion of Panx1 accelerated atherosclerosis progression, particularly in female *Apoe^−/−^* mice, and was associated with increased T-cell accumulation within atherosclerotic plaques, suggesting that Panx1 contributes to immune cell trafficking and lymphatic vessel function that protects against atherogenesis [68]. In addition, Panx1-mediated ATP release is an important regulator of endothelial purinergic signaling. ATP released through mechanosensitive Panx1 channels can activate endothelial Ca^2+^ signaling pathways that influence endothelial permeability, inflammatory responses, and leukocyte recruitment [69]. Although excessive extracellular ATP may promote vascular inflammation under certain conditions, physiological Panx1 signaling appears essential for maintaining vascular and lymphatic homeostasis [67,68].

### 5.7. Chloride Channel

Chloride (Cl^−^) channels are important regulators of endothelial membrane potential, cell volume, oxidative stress, inflammation, and vascular homeostasis. Chloride channels have been implicated in endothelial injury. Intracellular chloride channel 1 (CLIC1) expression is elevated in atherosclerotic lesions and mediates oxidative stress, inflammatory cytokine production, and endothelial activation. Pharmacological suppression of CLIC1 reduced reactive oxygen species generation, decreased expression of ICAM-1 and VCAM-1, and attenuated atherosclerotic lesion formation [70]. And CLIC4 promotes ox-LDL-induced endothelial apoptosis, inflammation, and oxidative stress, whereas inhibition of CLIC4 protects endothelial cells from atherogenic injury [71]. In contrast, activation of endothelial glycine-gated chloride channels (known as glycine receptors, GlyRs) was proposed to exert anti-atherogenic effects by hyperpolarizing endothelial cells, suppressing NADPH oxidase-mediated superoxide generation [72]. However, the roles of chloride channels in endothelial atherosclerotic signaling remain less well defined than those of calcium- and potassium-conducting channels, and additional studies are needed to clarify their endothelial specificity, molecular mechanisms, and translational relevance.

## 6. Discussion and Future Perspectives

Endothelial dysfunction is a hallmark of atherosclerosis and plays a critical role throughout disease initiation, progression, and plaque destabilization. Increasing evidence demonstrates that endothelial ion channels are not merely passive regulators of membrane excitability but active participants in vascular homeostasis, inflammatory signaling, mechanotransduction, metabolic sensing, and cell survival (Table 1). Ion channels have emerged as important modulators during atherogenesis [21,22,43,48,73,74,75,76]. They regulate key pathogenic processes such as calcium homeostasis, nitric oxide production, oxidative stress, leukocyte recruitment, endothelial permeability, apoptosis, ferroptosis, autophagy, and endothelial-to-mesenchymal transition.

### 6.1. Integrated Endothelial Bioelectrical Signaling Networks

**Figure 1:**
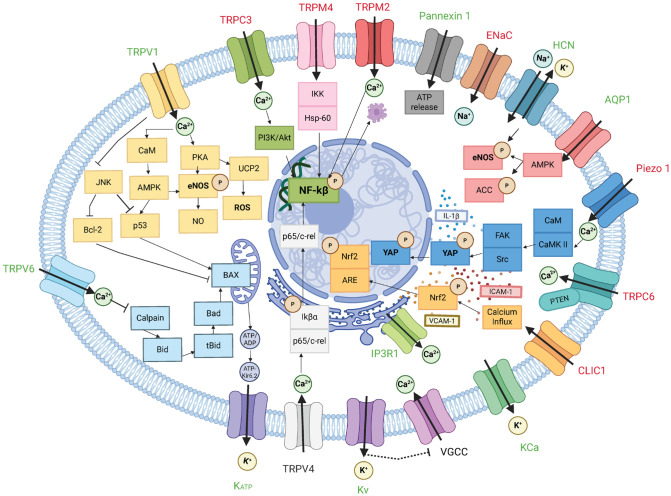
Summary of endothelial ion channel-mediated signal transduction pathways involved in atherosclerosis. Pathways labeled in red indicate predominantly pathogenic signaling associated with endothelial dysfunction, inflammation, oxidative stress, apoptosis, or impaired vasodilation, whereas pathways labeled in green indicate predominantly protective signaling associated with endothelial homeostasis, NO production, barrier preservation, or anti-inflammatory responses. Blue color dots refer to IL-1β, red refers to ICAM1, and orange refers to VCAM1.

Notably, endothelial ion channels exhibit diverse and sometimes opposing effects on atherosclerosis. Channels such as TRPV1, TRPV6, K_Ca_, K_ATP_, AQP1, IP3R1, and Pannexin 1 exhibit protective functions by supporting vasodilatory signaling, calcium homeostasis, barrier integrity, vascular homeostasis, and anti-inflammatory responses [28,29,40,41,42,43,44,67,68], whereas TRPC3, TRPC6, TRPM2, TRPM4, ENaC, Piezo1, and CLIC channels promote endothelial dysfunction and plaque progression [23,24,25,26,37,38,52,53,54,55,63,70,71], as shown in Figure 1. Despite their functional diversity, many of these channels converge on common signaling pathways involving intracellular Ca^2+^ dynamics, reactive oxygen species generation, endothelial nitric oxide synthase activity, and inflammatory transcriptional programs.

A central feature of these networks is the functional coupling between Ca^2+^ signaling and membrane potential. Calcium-permeable channels, including TRPC, TRPV, TRPM, Piezo1, and intracellular calcium-release channels such as IP3R1, shape the amplitude and duration of endothelial Ca^2+^ signals [20,21,23,24,26,28,31,33,34,35,37,52,54,55,57,75]. These signals may activate protective pathways such as eNOS-dependent NO production, but excessive or sustained Ca^2+^ signaling can promote NF-κB activation, YAP/TAZ signaling, inflammasome activation, apoptosis, EndMT, and endothelial dysfunction. Potassium channels provide a complementary regulatory layer by controlling membrane potential. Activation of K_Ca_, K_ATP_, and K_v_ channels promotes endothelial hyperpolarization, supports Ca^2+^ entry, enhances eNOS activity, and preserves endothelium-dependent relaxation [40,41,43,44,46]. Thus, Ca^2+^-conducting channels and K^+^ channels operate as interconnected modules that jointly regulate endothelial homeostasis.

Mechanotransduction further illustrates the network behavior of endothelial ion channels. Piezo1 and TRPV4 convert hemodynamic and mechanical forces into Ca^2+^-dependent signaling responses, but their effects depend strongly on vascular context. Under physiological shear stress, mechanosensitive signaling may support endothelial homeostasis, whereas under disturbed flow, oscillatory shear stress, ox-LDL exposure, or plaque-associated mechanical stress, Piezo1 and TRPV4 can activate YAP/TAZ, NF-κB, inflammasome signaling, EndMT, pyroptosis, and plaque vulnerability [32,33,35,52,53,54,55,56,57,58]. Together, these findings support a systems-level model in which endothelial ion channels and transporters coordinate vascular homeostasis and atherogenesis through convergent bioelectrical signaling networks rather than isolated linear pathways.

### 6.2. Redox Regulation and Oxidative Stress in Endothelial Ion Channel Signaling

Closely linked to integrated endothelial bioelectrical networks is the reciprocal relationship between ion channel activity and redox signaling. Under physiological conditions, low levels of reactive oxygen species (ROS) contribute to adaptive endothelial signaling, whereas excessive or sustained ROS production disrupts calcium homeostasis, reduces nitric oxide (NO) bioavailability, activates inflammatory pathways, and promotes endothelial dysfunction [77]. Endothelial ion channels and transporters influence this balance by regulating Ca^2+^ influx, membrane potential, mitochondrial function, NADPH oxidase activity, eNOS signaling, and inflammatory mediator expression [23,24,26,29,42,44,55,59].

Several channel families illustrate this redox connection. TRPC3-dependent Ca^2+^ influx promotes NF-κB activation, VCAM-1 expression, monocyte adhesion, ER stress, apoptosis, and endothelial dysfunction under hyperlipidemic conditions [23,24,25], while TRPC6 contributes to ox-LDL-induced mitochondrial apoptotic signaling and endothelial injury [26]. TRPM2 deficiency reduces vascular ROS accumulation, inflammatory factor expression, and plaque formation in hypercholesterolemic models [37]. TRPM4, ENaC, and CLIC channels have also been linked to ox-LDL-induced injury, inflammatory activation, oxidative stress, and plaque progression [63,70,71]. Conversely, several channels appear to preserve endothelial redox homeostasis. TRPV1 and TRPV6 limit endothelial inflammation, senescence, apoptosis, and ox-LDL-induced injury [28,29,36]. KCa, KATP, and Kv channels support membrane hyperpolarization, eNOS activation, NO production, and endothelium-dependent relaxation, thereby counteracting oxidative endothelial dysfunction. Together, these findings suggest that endothelial ion channels and transporters regulate oxidative stress through interconnected feedback loops involving Ca^2+^ dynamics, membrane potential, NO signaling, ROS generation, mitochondrial function, and inflammatory activation.

### 6.3. Challenges and Future Directions in Endothelial Ion Channel Research

Although growing evidence highlights the importance of endothelial ion channels in atherosclerosis, several challenges remain for the field.

First, some endothelial ion channels’ function remains unclear in atherosclerosis despite their crucial roles in vascular biology. Voltage-gated sodium (Na_v_) channels have been identified in endothelial cells and have been implicated in angiogenesis, endothelial migration, and barrier regulation, yet their contributions to endothelial inflammation and plaque development remain largely unknown. Similarly, voltage-gated calcium (Ca_v_) channels have long been associated with the anti-atherosclerotic effects of calcium channel blockers, but the specific functions of endothelial Ca_v_ channel subtypes in vascular inflammation and endothelial signaling have not been systematically investigated. Other channels, including two-pore domain potassium channels and store-operated calcium channels, may also participate in endothelial dysfunction but remain to be characterized in atherosclerotic disease.

Second, the dynamic regulation of ion channel expression, localization, and activity throughout different stages of atherosclerosis remains unexplored. Atherosclerotic lesions evolve through distinct phases characterized by endothelial activation, inflammatory cell recruitment, lipid accumulation, fibrous cap formation, and plaque rupture, yet little is known about how endothelial ion channel networks are remodeled during these transitions.

Third, interactions among multiple ion channels and their integration with metabolic, mechanical, inflammatory, and epigenetic signals require further investigation. Endothelial cells express a complex repertoire of ion channels that function cooperatively rather than independently. For example, mechanosensitive channels such as Piezo1 interact with TRP channels and potassium channels to coordinate endothelial responses to disturbed flow. Defining these bioelectrical signaling networks will be essential for understanding endothelial dysfunction in atherosclerosis.

Last, a major strength of the current literature is the use of complementary in vitro, genetic, pharmacological, and mouse-model approaches, which have provided important mechanistic insight into calcium signaling, membrane potential regulation, oxidative stress, inflammation, and endothelial survival. However, much of the evidence is derived from cultured endothelial cells and atherosclerosis-prone mouse models such as *Apoe^−/−^* and *Ldlr^−/−^* mice. In vitro systems allow controlled mechanistic studies but do not fully reproduce endothelial heterogeneity, chronic hemodynamic forces, immune-cell interactions, or the plaque microenvironment. Conversely, in vivo models provide valuable preclinical evidence but may be influenced by species differences, vascular-bed specificity, diet, sex, age, systemic lipid levels, immune responses, and non-endothelial effects of pharmacological or genetic interventions. Reproducibility across models also remains an important issue, as the same channel may exert protective or pathogenic effects depending on disease stage, mechanical environment, and cellular context. Therefore, current findings should generally be interpreted as experimental or preclinical evidence rather than clinically validated conclusions.

Emerging technologies may help define how endothelial ion channels and transporters are regulated across vascular beds, disease stages, and plaque microenvironments. Single-cell atlases of endothelial heterogeneity can identify which ion channel subtypes are enriched in protective, inflamed, senescent, angiogenic, or EndMT-like endothelial populations, thereby clarifying whether specific channels are associated with adaptive or dysfunctional endothelial states. Spatial transcriptomics of human plaques can further map channel expression to anatomically and functionally distinct regions, such as the fibrous cap, plaque shoulder, intraplaque neovessels, and inflammatory cell-rich areas, linking ion channel regulation to local mechanical stress, lipid accumulation, hypoxia, inflammation, and plaque vulnerability. Endothelial cell-specific genetic models and CRISPR-based perturbation approaches will be important for determining whether changes in channel expression or activity are causal drivers of endothelial dysfunction rather than secondary markers of vascular injury. In parallel, selective pharmacological modulators and nanoparticle-based delivery systems may enable more precise targeting of endothelial ion channels while minimizing off-target effects in the heart, nervous system, kidney, immune cells, and vascular smooth muscle. Finally, AI-assisted integration of transcriptomic, proteomic, electrophysiological, imaging, and pharmacological datasets may help identify channel interaction networks, predict context-dependent channel functions, and support precision medicine strategies aimed at restoring endothelial calcium homeostasis, membrane potential regulation, redox balance, and inflammatory control in atherosclerotic cardiovascular disease.

## 7. Conclusions

In conclusion, endothelial ion channels and transporters play important roles in atherosclerosis by regulating Ca^2+^ signaling, membrane potential, nitric oxide bioavailability, redox balance, mechanotransduction, and inflammation. These channels act as interconnected bioelectrical signaling modules that integrate vascular stimuli and shape endothelial function. Although current evidence supports their relevance in endothelial dysfunction and plaque progression, most findings remain experimental or preclinical. Further human validation and endothelial-specific therapeutic strategies are needed to translate these mechanisms into clinically useful approaches for atherosclerotic cardiovascular disease.

## Figures and Tables

**Table 1:** Endothelial ion channels in atherosclerosis.

Endothelial Channel	Protective or Pathogenic	Major Role in Atherosclerosis	Major Mechanism(s)	Potential Therapeutic Strategy	Evidence Level
TRPC3	Pathogenic	Promotes endothelial activation and plaque progression	NF-κB activation, VCAM-1 expression, monocyte adhesion, ER stress, apoptosis [23,24,25]	TRPC3 inhibition	In vitro + *Apoe*^−/−^ mouse
TRPC6	Pathogenic	Promotes endothelial injury and lesion progression	Ox-LDL-induced apoptosis, mitochondrial dysfunction [26,27]	TRPC6 inhibition; miR-26a restoration	In vitro + preclinical
TRPV1	Protective	Suppresses endothelial dysfunction and vascular inflammation	Inhibits ROCK signaling, reduces endothelial microparticles [29], suppresses senescence (ISG15-p53 pathway) [30]	TRPV1 activation	In vitro + preclinical
TRPV4	Predominantly Protective *	Regulates endothelial homeostasis and mechanotransduction	eNOS activation, NO production [32], EndMT regulation, mechanosensing [33,34]	Context-dependent modulation	In vitro + preclinical; context-dependent;
TRPV6	Protective	Reduces endothelial apoptosis and inflammation	PKA/UCP2 signaling, suppression of TNF-α, IL-1β, IL-6 [36]	TRPV6 activation	In vitro + *Apoe^−/−^* mouse
TRPM2	Pathogenic	Promotes oxidative stress and vascular inflammation	ROS sensing, Ca^2+^ influx, inflammatory signaling [37]	TRPM2 inhibition	In vitro + preclinical
TRPM4	Pathogenic	Contributes to endothelial dysfunction and plaque instability	Autophagy, apoptosis, necrotic core formation [38]	TRPM4 inhibition	preclinical + pharmacological inhibition studied
K_Ca_2.3/K_Ca_3.1	Protective	Preserves endothelial function and vasodilation	Endothelial NO signaling	K_Ca_ activators (e.g., SKA-31) [42]	Preclinical
K_ATP_ (K_ir_6.1/SUR)	Protective	Protects against lesion development and endothelial dysfunction	Metabolic sensing, endothelial NO production [44]	K_ATP_ activation	*Apoe^−/−^* mouse
K_v_ channels	Protective	Maintain endothelial relaxation under atherogenic conditions	Hyperpolarization, Ca^2+^ signaling, NO production [40,46]	K_v_ channel activation	Preclinical vessel-function evidence
Piezo1	Pathogenic	Drives endothelial inflammation and plaque progression	Mechanotransduction, Ca^2+^ influx, YAP/TAZ, NF-κB signaling [55,56]	Piezo1 inhibition	In vitro + pre-clinical
AQP1	Protective	Maintains endothelial integrity and plaque stability	Water transport, barrier function	AQP1 activation [61]	Human transcriptomic association + in vitro
AQP5	Pathogenic (indirect evidence)	Associated with inflammation	Inflammatory signaling [62]	Further investigation needed	Preclinical
ENaC	Pathogenic	Promotes endothelial dysfunction and vascular inflammation	Endothelial stiffening, cytokine production, and adhesion molecule expression	ENaC blockers (amiloride [7], benzamil [63])	In vitro + preclinical LDLr^−/−^ mouse
HCN (If)	Protective (indirect evidence)	Improves endothelial function and reduces lesion burden	Enhanced eNOS activity, reduced inflammation [64]	If-channel inhibition (ivabradine)	Preclinical
IP3R1	Protective	Maintains endothelial Ca^2+^ homeostasis and suppresses inflammation	Intracellular Ca^2+^ release, reduced VCAM-1/ICAM-1 expression [66]	Stabilization of IP3R1	Endothelial-specific preclinical
Pannexin 1	Protective	Limits plaque development and supports vascular homeostasis	ATP release, purinergic signaling, and immune regulation [67,68]	Preservation of Panx1 signaling	*Apoe^−/−^* mouse
CLIC1	Pathogenic	Promotes endothelial activation and inflammation	VCAM-1/ICAM-1 expression [70]	CLIC1 inhibition	In vitro + *Apoe^−/−^* mouse
CLIC4	Pathogenic	Promotes endothelial apoptosis and oxidative injury	Ox-LDL-induced apoptosis and inflammation [71]	CLIC4 inhibition	In vitro
GlyR	Protective (indirect evidence)	Suppresses endothelial oxidative stress	Hyperpolarization, reduced NADPH oxidase activity [72]	Glycine/GlyR activation	Hypothesis evidence

* TRPV4 exhibits context-dependent effects, with both atheroprotective and pro-inflammatory roles reported depending on mechanical and inflammatory stimuli.

## Data Availability

No new data were created or analyzed in this study. Data sharing is not applicable to this article.

## Abbreviations

The following abbreviations are used in this manuscript:

TRP	Transient receptor potential
TRPC	Transient receptor potential canonical
TRPM	Transient receptor potential melastatin
TRPML	Transient receptor potential mucolipin
TRPV	Transient receptor potential vanilloid
K_ca_	Calcium-activated potassium channel
K_ATP_	ATP-sensitive potassium channel
K_v_	Voltage-gated potassium channel
Ca_v_	Voltage-gated calcium channel
Na_v_	Voltage-gated sodium channel
AQP	Aquaporin
ENaC	Epithelial sodium channel
HCN	Hyperpolarization-activated cyclic nucleotide-gated channel
IP3R1	Inositol 1,4,5-trisphosphate receptor 1
Panx1	Pannexin 1
GlyR	Glycine receptor, Glycine-gated chloride channels
SUR	sulfonylurea receptor
I_f_	Pacemaker current
4-AP-4	4-aminopyridine
ox-LDL	oxidized low-density lipoprotein
EndMT	Endothelial-to-mesenchymal transition
VCAM-1	vascular cell adhesion molecule-1
ISG15	Interferon-stimulated gene 15
eNOS	endothelial nitric oxide synthase
HUVEC	Human umbilical vein endothelial cell
ROS	Reactive oxygen species
NO	Nitric oxide
